# Molecular characterization and genetic variability of *Toxocara vitulorum* from naturally infected buffalo calves for the first time in Bangladesh

**DOI:** 10.1017/S0031182024000842

**Published:** 2024-07

**Authors:** Hiranmoy Biswas, Nurnabi Ahmed, Babul Chandra Roy, Mohammad Manjurul Hasan, MD Khalilur Rahman, Md. Hasanuzzaman Talukder

**Affiliations:** 1 Department of Parasitology, Bangladesh Agricultural University, Mymensingh, Bangladesh; 2 Department of Livestock Services, Dhaka, Bangladesh

**Keywords:** Bangladesh, Buffalo calves, Genetic variability, Phylogeny, SNP, *T. vitulorum*

## Abstract

*Toxocara vitulorum* is one of the deadliest parasite of buffalo calves in Bangladesh. This study was conducted to explore genetic variability within and among the *T. vitulorum* populations in buffalo calves of Bangladesh. Genomic DNA was extracted, *ITS2, COX1* and *NAD1* gene were amplified and sequenced. Distinct 29 *ITS2*, 21 unique *NAD1* and 24 *COX1* genotypes were detected among the *T. vitulorum* of different geographic regions. These three gene genotypes similarities ranged from 97 to 99%, when these were compared to best hit scoring *T. vitulorum* sequences retrieved from GenBank. A total of 12 and 6 unique haplotypes were detected for *COX1* and *NAD1* gene sequences. The average nucleotide and haplotype diversity for *COX1* and *NAD1* were 0.0931 & 0.89493 and 0.00658 & 0.77895 respectively and the recorded values were more dispersed than previously published values. The pairwise Nst values ranged from −0.050 to 0.602 and Fst from −0.050 to 0.600 between all the *T. vitulorum* genotypes indicated huge genetic differentiation which were reportedly higher than other published reports Fst values. This is the first report of *T. vitulorum* on the basis of *COX1* gene in Bangladesh. The study findings will be helpful for further extensive epidemiological studies regarding anthelmintic resistance, control and prevention of *T. vitulorum* infection in buffalo calves.

## Graphical abstract

**Figure:**
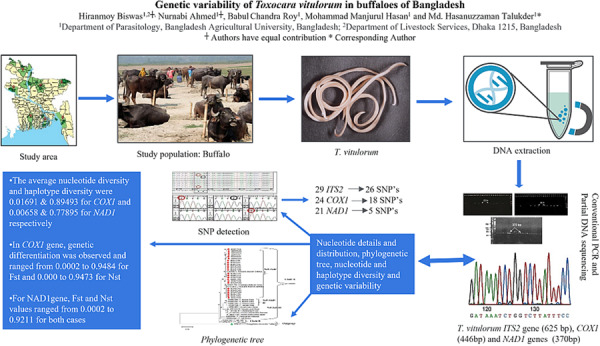

## Introduction

*Toxocara vitulorum* is one of the deadliest ascarid nematode that lives in the small intestine of cattle, buffalo and other bovids (Biswas *et al*., 2022). It has worldwide distribution but mostly found in bovids of tropical and subtropical regions (Dorny *et al*., 2015). This zoonotic ascarid causes visceral larval migrans (VLM) in humans (Toochukwu, 2017). In the young buffalo calves aged under 3 months, toxocariasis is one of the prime causes of morbidity and mortality (Rahman *et al*., 2018; Biswas *et al*., 2022). The most common routes of receiving infection by this buffalo calves are transmammary and transplacental route while they can also get infection by consumption of larvated eggs contaminated forage (Rast *et al*., 2013). In buffalo, the prevalence of *T. vitulorum* varied from 2 to 32% while 11 to 57% for buffalo calves in Bangladesh for last 12 years and it is still increasing with the course of time (Islam *et al*., 2005; Rahman *et al*., 2018). Previous reports also unveiled that the buffalo calves had 3 times higher odds of getting infection than dam buffalo (Rahman *et al*., 2018). It is assumed that if this parasitic infection is not controlled properly, the prevalence and mortality can be reached up to 100 and 50% in buffalo calves respectively (Radostits *et al*., 2006; Rast *et al*., 2013). Continuous reporting of ineffectiveness of benzimidazole s against *T. vitulorum* from different buffalo rearing farms and *Bathan* (a vast waking marshy green grass land on the river bed) regions of Bangladesh have been recorded for last 12 years and developing resistance against some available benzimidazoles such as albendazole and levamisole (Biswas *et al*., 2022). It is evident that parasitic nematodes are incredibly diverse and treatments rely on a limited arsenal of anthelmintic drugs with same drug classes, over-reliance and inappropriate use of these anthelmintics have placed strong selective pressures on parasites and caused the evolution of anthelmintic resistance (AR) to every drug class (Kotze *et al*., 2020). In many cases it has been proved that the evolution of AR to every drug class depend on genetics of resistant nematode, therefore phenotypic variation in anthelmintic responses that can be explained by genetic variation in a population (Evans *et al*., 2021). As of today, few studies were conducted to determine the prevalence and associated factors of transmission of *T. vitulorum* in Bangladesh, but interestingly no molecular based approach taken into consideration to characterize the ascarid which will be a tool to unveil resources for understanding the pathogen ecology, epidemiology and control (Halajian *et al*., 2010; Sultan *et al*., 2015). In pursuit of drawing the hidden relationship between AR and genetic makeup of *T. vitulorum,* we ought to unveil molecular data from the circulating *T. vitulorum* in Bangladesh as first step. Currently, a wide range of molecular techniques such as PCR, restriction fragment length polymorphism, randomized amplified polymorphism DNA and sequencing have been used widely to identify parasite species more precisely (Prichard and Tait, 2001; Ahmed *et al*., 2023). The nuclear ribosomal DNA (rDNA) particularly internal transcribed region 2 (*ITS2*) is a potential marker for identification of species because of its some distinct attributes such as easy amplification, integrity of conserved regions, fast evolution of variable nuclear loci, good number of rRNA clusters to unleash closely interlinked species (Li *et al*., 2006; Chen *et al*., 2012; Ahmed *et al*., 2023). The mitochondrial nicotinamide dehydrogenase subunit 1 gene (*NAD1*) and cytochrome oxidase 1 (*COX1*) genes have been used as candidates for studying diversity and finding out population structure for a long time (Jones *et al*., 2009; Wickramasinghe *et al*., 2009*a*, 2009*b*). Therefore, we selected conventional PCR technique to amplify the *ITS2*, *COX1*, and *NAD1* gene markers followed sanger sequencing to detect *Toxocara* nematode at species level and finding similarities and dissimilarities among the identified isolates and with other isolates detected from other parts of the world base on genetic P distance. The investigation also established population structure and phylogenetic link between the detected *T. vitulorum* isolate and additional *T. vitulorum* isolates, as well as congenerics from distantly related species.

## Materials and methods

### Study area

**Figure 1.**
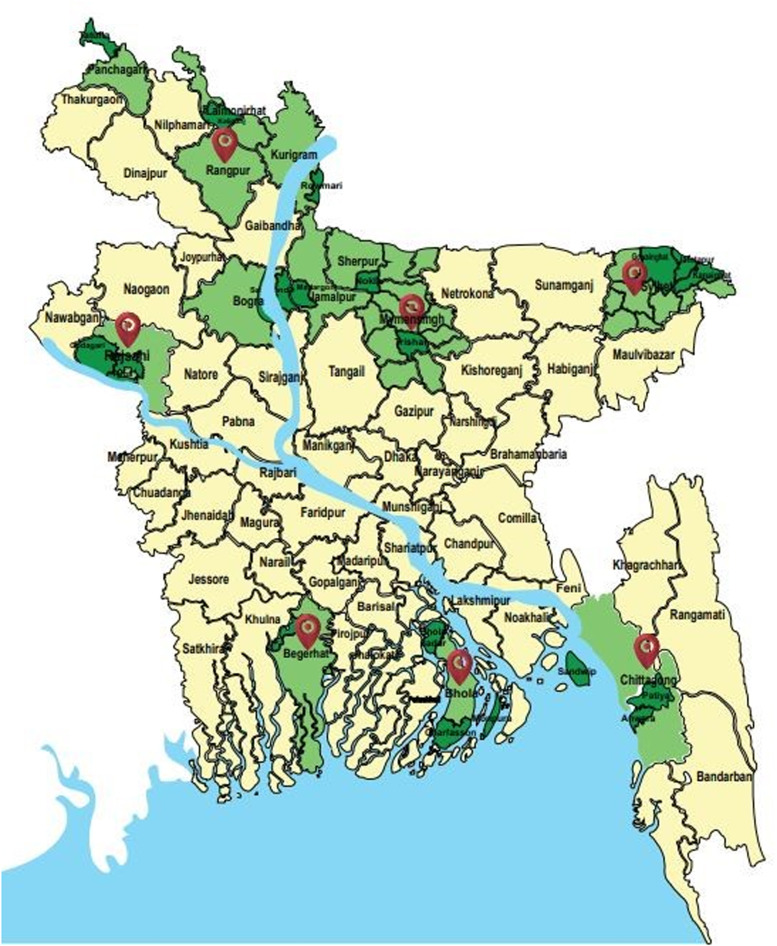
Map of Bangladesh showing the 7 study areas as Barishal, Chattogram, Khulna, Rajshahi, Rangpur, Mymensingh and Sylhet division.

The study was conducted on multiple sites of 7 divisions such as Barishal (Charfassion, Bhola Sadar in Bhola district of Barishal), Chattogram (Sandwip and Anowara upazila in Chattogram), Khulna (Fakirhat, Mongla and Morelgonj in Bagerhat district), Rajshahi (Godagari and Paba in Rajshahi district and Sariakandi in Bogura district), Rangpur (Kurigram Sadar, Rawmari in Kurigram district and Kaligonj in Lalmonirhat district), Mymensingh (Trishal, Madargonj and Nokla in Myemsingh, Jamalpur and Sherpur district, respectively) and Sylhet (Gowainghat, Jaintapur and Kanaighat in Sylhet district) during July 2018 to December 2020 (Fig. 1). These selecting areas were highly prevalent for toxocariasis and the availability of different age groups buffaloes (Rahman *et al*., 2018).

### Parasite collection

Based on the coproscopic examination, we selected buffalo calves harbouring *Toxocara* worms. We gave them anthelmintic treatments such as Ivermectin @0.2 mg kg^−1^ BWT (Biswas *et al*., 2022) and following treatment adult *Toxocara* worms were expelled with feces. Then these adult *Toxocara* worms were washed with normal saline and transported to the Department of Parasitology, Bangladesh Agricultural University for microscopic detection and then stored at −20°C for molecular investigation.

### Isolation of genomic DNA

Genomic DNA was extracted from 84 adult *Toxocara* species (12 from each division) by using QIA amp mini kit (Original product by Qiagen AG, Hombrechtikon, Switzerland and provided by Qiagen India Pvt. Ltd. Jasola, Delhi) according to manufacturer's recommendations (Khademvatan *et al*., 2013). The eluted DNA was stored at −20°C prior to PCR.

### Amplification of *ITS2*, *NAD1* and *COX1* gene and gel electrophoresis

#### *ITS2* (~625 bp)

*ITS2* gene was amplified from genomic DNA by using the conserved oligo-nucleotide primer pair: 3S (forward: 5′-CGGTGGATCACTCGGCTCGT-3) and 28A (reverse: 5′-CCTGGTTAGTTTCTTTTCCTCCGC-3′) (Wickramasinghe *et al*., 2009*a*, 2009*b*; Mahdy *et al*., 2020). PCR reaction with a final reaction volume of 25 μL was conducted. 5 μL of DNA extract, 12.5 μL of Taq® Green Master Mix, 5.5 μL of nuclease-free water, and 1 μL of each forward and reverse primer were used in the amplifications. The amplification programme was run in a MyCyclerTM heat cycler (BioRad, USA) with a 3 minutes initial denaturation at 94°C, 31 cycles of 30 s at 94°C, 30 s at 46°C, and 1 min at 72°C. For all primers, except the 12S and *ITS2* primers, which were annealed at 50 and 53°C, respectively, there was a final 5 min of extension at 72°C (Mahdy *et al*., 2020). The resultant gel was analysed and captured on camera using a transilluminator.

#### *NAD1* (~370 bp)

During the conventional PCR, the primer pairs (forward: 5′-TTCTTATGAGATTGCTTTT-3′ and reverse: 5′-TATCATAACGAAAACGAGG-3′) were used (Li *et al*., 2016; El-Seify *et al*., 2021). 9.75 μL autoclaved, distilled water, 5 μL PCR buffer (10×), 0.25 μL Taq, 2 μL dNTPs (2.0 mm), 1 μL DNA, 3 μL MgCl2 (25 mm) and 2 μL of each forward and reverse primer (working concentration: 10 μmol L^−1^) were all included in the PCR mixture in a 25 μL reaction volume. After a heated start of 94°C for 5 min and concluding with 72°C for 5 min, each of the 40 PCR cycles included 94°C for 30 s, 50°C for 30 s, and 72°C for 1 min (Li *et al*., 2016; El-Seify *et al*., 2021). The PCR products were observed using a UV transilluminator after being separated on 1% agarose gels and stained with ethidium bromide.

#### *COX1* (~446 bp)

A fragment of *COX1* was amplified by PCR, yielding a 446 bp sequence using primers JB3 (5′-T TTTTTGGGCATCCTGAGGTTTAT-3′) and JB4.5 (5′-TAAAGAAAGAACATAATGAAAATG-3′), a final volume of 25 μL was used for the PCR, which contained 7.5 μL of sterile distilled water that was free of RNase and DNase, 10 μL of 5× MyTaq Reaction buffer, 1 μL of each primer (20 pmol), 5 μL of template DNA (100–200 ng), and 0.5 μL of TaqDNA polymerase (1.25 IU)^4^ (Wickramasinghe *et al*., 2009*a*, 2009*b*;Oguz, 2018). The following were the conditions for the PCR: 5 minutes at 94°C for initial denaturation, 35 cycles of 30 s at 94°C, 45 s at 50°C, 35 s at 72°C, and 10 min at 72°C for the final extension. The PCR products were observed using a UV transilluminator after being separated on 1.5% agarose gels and stained with ethidium bromide (Oguz, 2018).

### PCR positive electrophoresis product purification

Purification of the *ITS2, COX1* and *NAD1* PCR electrophoresis products was accomplished with the use of SV Gel and PCR Clean Up System (Cat. No. A9281; Origin: Promega, USA). Purified products were sequenced using an Applied Biosystems automated DNA sequencer (3730 XL; Applied Biosystems, Foster City, USA) in accordance with manufacturer instructions from a commercial source (DNA Laboratories Sdn Bhd (736763-T), UKM-MTDC Technology Centre, Selangor, Malaysia through Invent Technology Ltd. Banani, Dhaka). The forward and reverse PCR primers orientation was same for both the forward and reverse reads.

### Intra-population diversity parameters and phylogeny

Using BLAST (https://blast.ncbi.nlm.nih.gov/Blast.cgi), unique sequences for each marker (29 *ITS2*, 21 *NAD1* and 24 *COX1*) generated in this study were compared with best hit scoring sequences available in GenBank (Supplementary Table S4). The Mega11 software was used to aligned the sequences using ClustalW program by using a gap opening penalty of 15.00 and gap extension penalty of 6.66 for both pairwise and multiple alignments as described by Nehra *et al*. (2022). Pairwise comparisons were made using the GenBank-retrieved sequences, and the BioEdit program (version 7.0.5.3) (https://bioedit.software.informer.com/7.2/) was utilized to determine similarities (%) (Ahmed *et al.,* 2023). Haplotype diversity, the average number of nucleotide change, and nucleotide diversity were among the characteristics linked to intra-population diversity that were measured using DnaSP version 5.1 (Rozas, 2017). After trimming every sequence at both ends, the phylogenetic analysis was carried out using the neighbor-joining method with the Tamura Nei parameter of evolution based on lowest BIC score (Bayesian Information Criteria) and AICc value (Akaike Information Criteria) in the Mega11 programme (Tamura *et al*., 2021). While MEGA v.11.0's default values were used to acquire the other settings, 1000 replicates were used to determine the bootstrap parameters for the definition of nodes statistical support (Tamura *et al*., 2013).

### Population genetic structure by using mitochondrial COX1 sequences

Genetic differences were estimated using statistics based on haplotypes (Hs), nucleotide sequences (Ks) and some other parameters such as average number of nucleotide differences in pairs (Kxy), genetic differentiation index based on the frequency of haplotypes (Gst), nucleotide-based statistics (Nst), nucleotide substitutions per site (Dxy) and net nucleotide substitutions per site (Da) using DnaSP ver. 6.12.03 (Rozas *et al*., 2017) the population pairwise genetic difference (Fst) to determine the genetic differentiation and population genetic structure.

## Results

### Morphological findings

**Figure 2.**
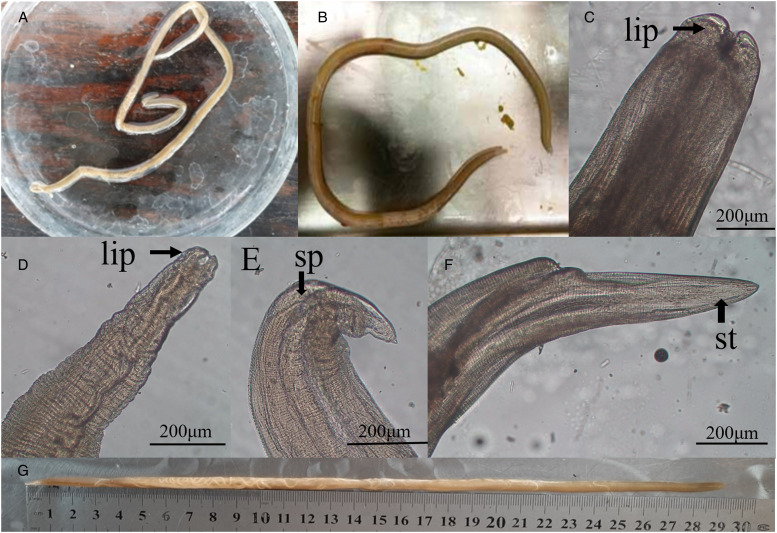
(A, B) *Toxocara vitulorum* collected from calves. (C, D) Anterior end of *T. vitulorum* showed 3 lips of male and female worm (lip), (E) posterior end of male showed coiled tail (black arrow) and showed spicules (sp) (F) posterior end of female showed short tail (st), and posterior end of female worm showed a straight tail end (long white arrow), (G) 30 cm long female *T. vitulorum*.

*Toxocara vitulorum* is a large, robust worm up to 30 cm long with three large, prominent lips. The body was soft and translucent with clearly cuticle (Fig. 2A and B). The mean length of male and female parasite was 18.5 (±1.2 cm) and 24.20 (±6.2 cm) 29.11 cm) and mean width were 0.4 and 0.6 mm in respectively. The three well defined lips; two sub ventral and one dorsal lip were determined from the worms (Fig. 2C and D). The male worms had a posterior end curved ventrally and posterior end exhibited two spicules (Fig. 2E). While, in female posterior end was distinguishable a straight-tailed (Fig. 2F).

### Species identification and genotyping

**Figure 3.**
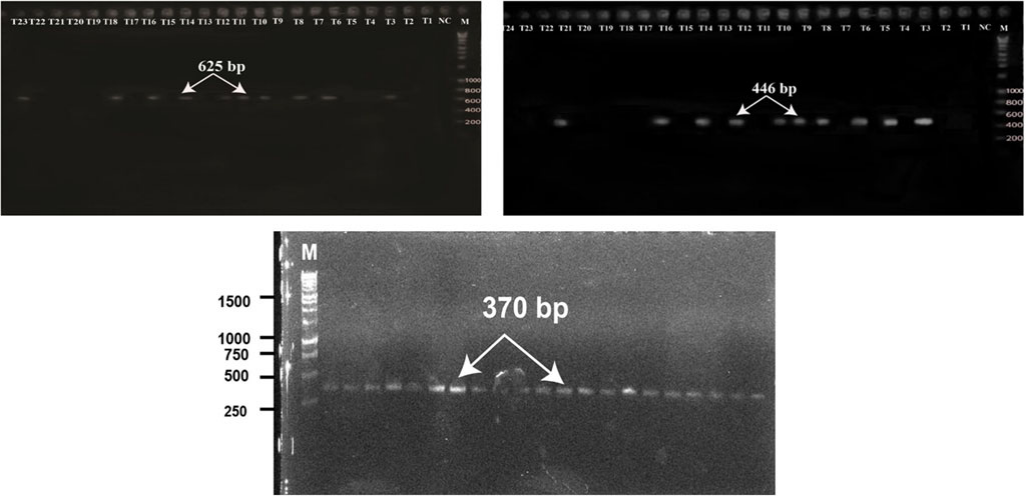
Conventional PCR product showing a. *T. vitulorum ITS2* gene (625 bp), b. *COX1* (446 bp) and c. *NAD1* genes (370 bp) in separate agarose gel. [Lane M (Marker-1 kb), lane NC (negative control)].

To validate the species of *T. vitulorum*, the *ITS2, COX1* and *NAD1* gene were amplified from 84 samples from seven divisions of Bangladesh and conventional PCR product showing *T. vitulorum ITS2* gene (625 bp), *COX1* (446 bp) and *NAD1* genes (370 bp) in separate agarose gel (Fig. 3).

From 84 positive PCR products, 84 *ITS2,* 79 for *COX1* and 74 for *NAD1* sequences were produced. Unfortunately, sequencing of 5 *T. vitulorum* isolates for *COX1* and *NAD1* was failed to generate. On the contrary, 5 sequenced data for *NAD1* gene failed to generate due to numerous ambiguous nucleotides positioning. Among 84 *ITS2* sequences, 29 distinct genotypes were identified while 79 *COX1* and 74 *NAD1* sequences, 24 and 21 distinct genotypes were found.

When compared to five *ITS2* reference sequences of *T. vitulorum* (GenBank accession nos. MK100346.1, MG214152.1, FJ418784.1, MG214151.1 and KY442062.1), the newly generated *ITS2* genotype similarities ranged from 97 to 100%. Pairwise nucleotidic genetic distances (p-distance model) were measured for the partial *ITS2* sequences of *T. vitulorum* isolates in the present study with best hit scoring reference sequences of different countries retrieved from GenBank and genetic distances ranging from 0.000 to 0.0326 (Supplementary Table S1).

Twenty-six single nucleotide polymorphisms (SNPs) were found when 29 *ITS2* genotypes were aligned with the reference sequence (MK100346.1 in India). These SNPs resulted from substitutions at nucleotide positions 60, 66, 116, 124, 130, 132, 133, 135, 150, 156, 158, 164, 177, 192, 205, 206, 234, 274, 275, 277, 298, 301, 308, 317, 319 and 331. In such substitutions, there were six transitions (three T˂-˃C and three A<->G) and twenty transversions (seven A˂->T, two G<->C, five A˂-˃C, and six G<->T) (Table 1). Table 2 shows that the total haplotype diversity and nucleotide diversity of *T. vitulorum* among its *ITS2* sequences from seven divisions in Bangladesh were 0.83498 and 0.01530, respectively. Comparing the newly generated *COX1* and *NAD1* genotypes of *T. vitulorum* to the best hit scoring sequences from GenBank revealed matches ranging from 98 to 100% (Supplementary Tables S2 and S3). For the *COX1* and *NAD1* genes, respectively, 79 and 74 amplicons yielded 12 and 6 distinct haplotypes. When compared to the reference sequence (AJ920062.1) in the *COX1* gene, 18 SNPs were observed at positions 104, 14, 149, 155, 185, 197, 218, 220, 227, 245, 284, 296, 314, 344, 362, 368, 376 and 389 (Table 3). Five SNPs were found in the *NAD1* gene (T<->G, A<->T, and G<->A) (Table 4). SNP's were both transitions (A<->G, C<->T) and translations (<A->T, C<->G and G<->T) type in nature (Table 3). The *T. vitulorum* isolates from Bangladesh showed a high degree of diversity in the *COX1* and *NAD1* genes; for *COX1*, the average nucleotide diversity was 0.01691 and the haplotype diversity was 0.89493, while for *NAD1*, the average nucleotide diversity was 0.00658 and the haplotype diversity was 0.77895 (Table 2).

**Table 1.** Nucleotide details and distribution of 29 *ITS2* of *T. vitulorum* isolated from buffalo with reference sequence retrieved from GenBank (MK100346.1)

Genotypes	Nucleotide distribution																									
	60	66	116	124	130	132	133	135	150	156	158	164	177	192	205	206	234	274	275	277	298	301	308	317	319	331
MK100346.1*T. vitulorum_* Nagaland	C	G	T	T	T	T	T	G	A	A	T	A	A	A	T	A	A	A	A	G	T	A	G	C	A	T
BDBSTV01	.	.	.	.	.	.	.	.	.	.	.	.	.	.	.	.	.	.	.	.	.	.	.	.	.	.
BDBSTV05	.	.	.	.	.	.	.	.	.	.	.	.	.	.	.	.	.	.	.	.	.	.	.	.	.	.
BDBSTV08	.	.	.	.	.	.	.	.	.	C	.	.	.	.	.		C	.	.	T	.	.	.	.	.	.
BDBSTV12	.	.	.	.	.	.	.	.	.	C	.	.	.	.	.	T	C	G	T	T	.	.	.	.	.	.
BDCGTV01	.	.	.	.	.	.	.	.	T	.	.	.	.	.	A	.	.	.	.	.	.	.	.	.	.	.
BDCGTV02	.	.	.	.	.	.	.	.	T	.	.	.	.	.	A	.	.	.	.	.	.	.	.	.	.	.
BDCGTV05	.	T	.	.	.	G			T	.	.	.	.	.	A	.	.	.	.	.	.	.	.	.	.	.
BDCGTV07	.	.	.	.	.	.	.	.	T	.	.	.	.	.	A	.	.	.	.	.	.	.	.	.	.	.
BDKLTV08	.	.	.	.	.	.	.	.	.	.	.	.	.	.		.	.	.	.	.	.	.	.	.	.	.
BDKLTV01	.	.	.	.	.	.	.	.	.	.	.	.		.	.	.	.	.	.	.	.	.	.	.	.	.
BDKLTV02	.	.	.	.	.	.	.	.	.	.	.	.	T	.	.	.	.	.	.	.	.	.	.	.	.	.
BDKLTV06	.	.	.	.	.	.	.	.	.	.	.	.	.	.	.	.	.	.	.	.	.	.	.	.	.	.
BDMSTV01	G	.	G	C	.	.	.	C	.	.	.	.	.	.	.	.	.	.	.	.	.	G	A	A	.	.
BDMSTV03	G	.	G	C	.	.	.	C	.	.	.	.	.	.	.	.	.	.	.	.	.	G	A	A	.	.
BDMSTV04	G	.	G	C	.	.	.	C	.	.	.	.	.	.	.	.	.	.	.	.	.	G	A	A	.	.
BDMSTV08	G	.	G	C	.	.	.	C	.	.	.	.	.	.	.	.	.	.	.	.	.	G	A	A	.	.
BDMSTV09	G	.	G	C	.	.	.	C	.	.	.	.	.	.	.	.	.	.			C	G	A	A	T	
BDSLTV02	G	.	G	C	.	.	.	C	.	.	.	.	.	.	.	.	.	.	C	.	.	G	A	A	.	G
BDSLTV03	G	.	G	C	.	.	.	C	.	.	.	.	.	.	.	.	.	.	C	.	.	G	A	A	.	G
BDSLTV04	G	.	G	C	.	.	.	C	.	.	.	.		C	.	.	.		C	.	.	G	A	A	.	G
BDSLTV05	G	.	G	C	.	.	.	C	.	.		G	.	.	.	.	.	.	C	.	.	G	A	A	.	G
BDRSTV02	.	.	.	.	.	.	.	.	.	.	.	.	.	.	.	.	.	.	.	.	.	.	.	.	.	.
BDRSTV03	.	.	.	.	.	.	.	.	.	.	.	.	.	.	.	.	.	.	.	.	.	.	.	.	.	.
BDRSTV05	.	.	.	.	.	.	.	.	.	.	.	.	.	.	.	.	.	.	.	.	.	.	.	.	.	.
BDRSTV06	.	.	.	.	.	.	.	.	.	.	.	.	.	.	.	.	.	.	.	.	.	.	.	.	.	.
BDRPTV01	.	.	.	.	.	.	.	.	.	.	.	.	.	.	.	.	.	.	G	.	.	.	.	.	.	.
BDRPTV04	.	.	.	.	.	.	.	.	.	.	C		.	.	.	.	.	.	.	.	.	.	.	.	.	.
BDRPTV05	.	.	.	.	.	.	.	.	.	.	.	.	.	.	.	.	.	.	.	.	.	.	.	.	.	.
BDRPTV06	.	.	.	.	A		G	.	.	.	.	.	.	.	.	.	.	.	.	.	.	.	.	.	.	.

BD, Bangladesh; RP, Rangpur; RS, Rajshahi; CG, Chattogram; KL, Khulna; BS, Barishal; SL, Sylhet; MS, Mymensingh; TV, *Toxocara vitulorum*, the sur number was representative of isolate number.

**Table 2.** Nucleotide diversity and haplotype diversity of *ITS2*, *COX1* and *NAD1* gene sequences of *T. vitulorum* isolated from 7 different topographic regions of Bangladesh

Geographical locations	*ITS2* gene				*COX1* gene				*NAD1* gene			
	No of sequences	No of haplotypes	Haplotype diversity (Hd)	Nucleotide diversity (Nd)	No of sequences	No of haplotypes	Haplotype diversity (Hd)	Nucleotide diversity (Nd)	No of sequences	No of haplotypes	Haplotype diversity (Hd)	Haplotype diversity (Hd)
Khulna	12	2	0.50	0.00141	12	3	0.833	0.00749	10	1	0.00	0.00
Barishal	15	3	0.833	0.00992	14	1	0.00	0.00	13	1	0.00	0.00
Chattogram	12	2	0.500	0.00282	12	1	0.00	0.00	10	2	0.667	0.00208
Mymensingh	16	2	0.400	0.00226	15	2	0.50	0.0015	12	1	0.00	0.00
Sylhet	10	2	0.50	0.0028	10	2	0.667	0.00208	10	2	0.667	0.00208
Rajshahi	12	1	0.00	0.00	12	3	1.00	0.00499	11	1	0.00	0.00
Rangpur	12	4	0.833	0.00562	10	1	0.00	0.00	12	1	0.00	0.00
Overall	87	16	0.01530	0.83498	82	12	0.89493	0.01691	77	6	0.77895	0.00658

**Table 3.** Nucleotide details and distribution of 24 *T. vitulorum COX1* gene isolated from buffalo with reference sequence retrieved from GenBank (AJ920062.1)

Genotypes	Nucleotide distribution																	
	104	143	149	155	185	197	218	220	227	245	284	296	314	344	362	368	376	389
AJ920062.1*Toxocara vitulorum* Sri Lanka	T	G	G	A	A	G	T	A	A	T	C	A	T	G	A	G	T	C
BDBSTV01	.	.	.	.	.	.	.	.	.	.	.	.	.	.	.	.	.	.
BDRPTV01	.	C	.	.	.	.	.	.	.	.	.	.	.	.	.	.	.	.
BDRPTV04	.	C	.	.	.	.	.	.	.	.	.	.	.	.	.	.	.	.
BDRPTV05	.	C	.	.	.	.	.	.	.	.	.	.	.	.	.	.	.	.
BDCGTV01	.	.	.	.	.	.	.	.	.	.	.	.	.	.	.	.	.	.
BDBSTV08	.	.	.	.	.	.	.	.	.	.	.	.	.	.	.	.	.	.
BDRSTV05	.	.	.	.	.	.	.	.	.	.	.	.	.	.	.	.	G	.
BDCGTV02	.	.	.	G	.	.	.	.	.	.	.	.	.	.	.	.	.	.
BDRSTV02	.	.	.	G	.	.	.	.	.	.	.	.	.	.	.	.	.	.
BDRSTV03	.	.	.	.	.	.	.	.	.	.	.	.	A	.	.	.	.	.
BDBSTV05	.	.	.	.	.	.	.	.	.	.	.	.	.	.	.	.	.	.
BDKLTV01	.	.	.	.	.	.	.	.	.	.	.	.	.	.	G	.	.	.
BDKLTV08	.	.	.	.	.	.	.	.	.	.	.	.	.	C	G	.	.	.
BDBSTV12	.	.	.	.	.	.	.	.	.	.	.	.	.	.	.	.	.	.
BDSLTV03	.	.	A	.	G	A	C	.	G	C	T	G	.	.	.	A	.	T
BDSLTV02	.	.	A	.	G	A	C	.	G	C	T	G	.	.	.	A	.	T
BDMSTV03	.	.	A	.	G	A	C	.	G	C	T	G	.	.	.	A	.	T
BDCGTV05	.	.	.	.	.	.	.	.	.	.	.	.	.	.	.	.	.	.
BDKLTV02	A	.	.	C	.	.	.	.	.	.	.	.	.	.	.	.	.	.
BDKLTV06	A	.	.	C	.	.	.	.	.	.	.	.	.	.	.	.	.	.
BDMSTV01	.	.	A	.	G	A	C	.	G	C	T	G	.	.	.	A	.	T
BDSLTV04	.	.	A	.	G	C	C	.	G	C	C	G	.	.	.	A	.	T
BDMSTV09	.	.	A	.	G	A	C	.	G	C	C	G	.	.	.	A	.	T
BDMSTV08	.	.	A	.	G	A	C	T	G	C	C	G	.	.	.	A	.	T

BD, Bangladesh; RP, Rangpur; RS, Rajshahi; CG, Chattogram; KL, Khulna; BS, Barishal; SL, Sylhet; MS, Mymensingh; TV, *Toxocara vitulorum*, the sur number was representative of isolate number.

**Table 4.** Nucleotide details and distribution of 21 *T. vitulorum NADI* gene isolated from buffalo with reference sequence retrieved from GenBank (AJ920062.1)

Genotypes	Nucleotide distribution				
	40	83	121	157	265
AJ937266.1 *T. vitulorum* Sri Lanka	G	T	G	T	T
BDKHTV01	.	.	.	.	.
BDKHTV02	.	.	.	.	.
BDMSTV03	.	A	.	G	.
BDKHTV06	.	.	.	.	.
BDMSTV05	.	A	.	G	.
BDSLTV06	.	A	.	G	A
BDCTTV01	.	.	.	.	.
BDCGTV02	.	.	.	.	.
BDCGTV05	.	.	.	.	.
BDSLTV03	.	A	A	G	A
BDSLTV02	.	A	A	G	A
BDBSTV12	.	.	.	.	.
BDRSTV02	A	G	.	.	.
BDRSTV03	A	G	.	.	.
BDRSTV04	A	G	.	.	.
BDBSTV05	.	.	.	.	.
BDRPTV05	A	G	.	.	.
BDRPTV01	A	G	.	.	.
BDBSTV01	.	.	.	.	.
BDKHTV01	.	A	.	G	.
BDKHTV02	A	G	.	.	.

BD, Bangladesh; RP, Rangpur; RS, Rajshahi; CG, Chattogram; KL, Khulna; BS, Barishal; SL, Sylhet; MS, Mymensingh; TV, *Toxocara vitulorum*, the sur number was representative of isolate number.

### Phylogeny

**Figure 4.**
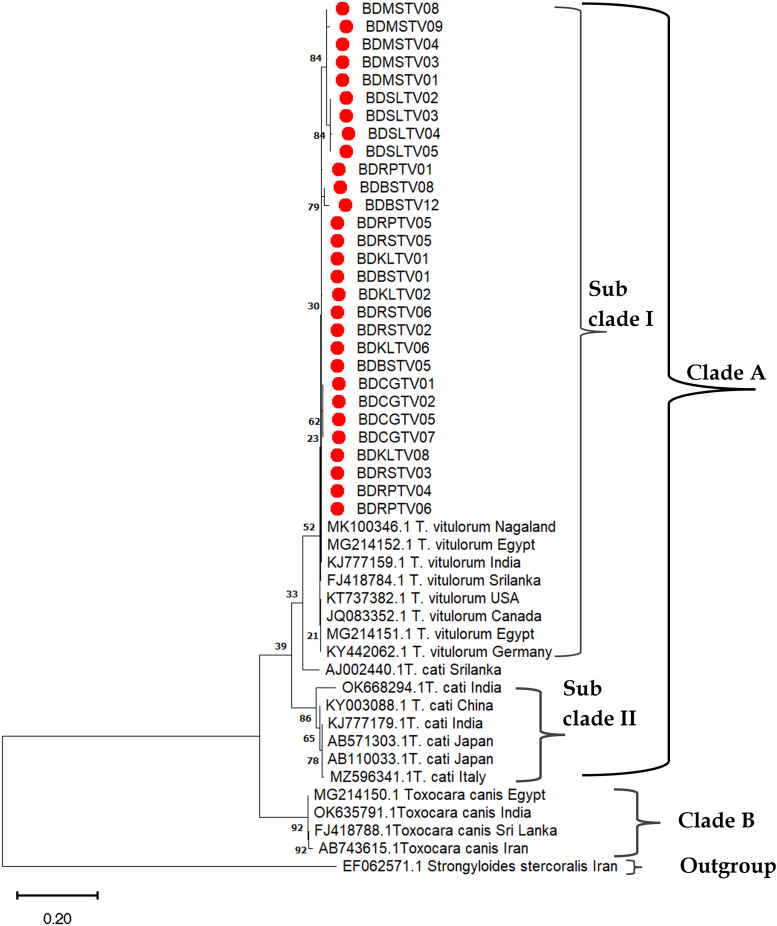
Neighbour-joining phylogenetic tree was constructed using partial *ITS2* gene of *T. vitulorum* isolates from different hosts and geographical regions. *Strongyloides stercoralis* was used as an out group. Red dots were study generated sequences. Scale bar indicates the proportion of sites changing along each branch. [BD, Bangladesh; RP, Rangpur; RS, Rajshahi; CG, Chattogram; KL, Khulna; BS, Barishal; SL, Sylhet; MS, Mymensingh; TV, *Toxocara vitulorum*, the sur number was representative of isolate number].

The phylogenetic tree has been constructed by 29 *ITS2* genotypes was divided into two clades: A and B. Furthermore, clade A was divided into subclades I and II, where *Strongyloides stercoralis* was used as outgroup. The neighbour-joining (NJ) phylogenetic tree demonstrated that *T. vitulorum* isolates clustered together with the reference sequences of Sri Lanka, USA, Canada, Egypt & Germany that belong to the subclade I under the clade A without any distinct geographical boundaries (Fig. 4).

In subclade II under clade A, the reference sequence of *T. cati* from China, India, Japan, Italy was clustered in same position of the tree, whereas, the reference sequences of *T. canis* from Egypt, India, Sri Lanka, Iran under clade B were grouped in same position. These findings unveiled that different species of *Toxocara* nematode has different genomic background and thus *Toxocara* species with same genomic background were clustered.

**Figure 5.**
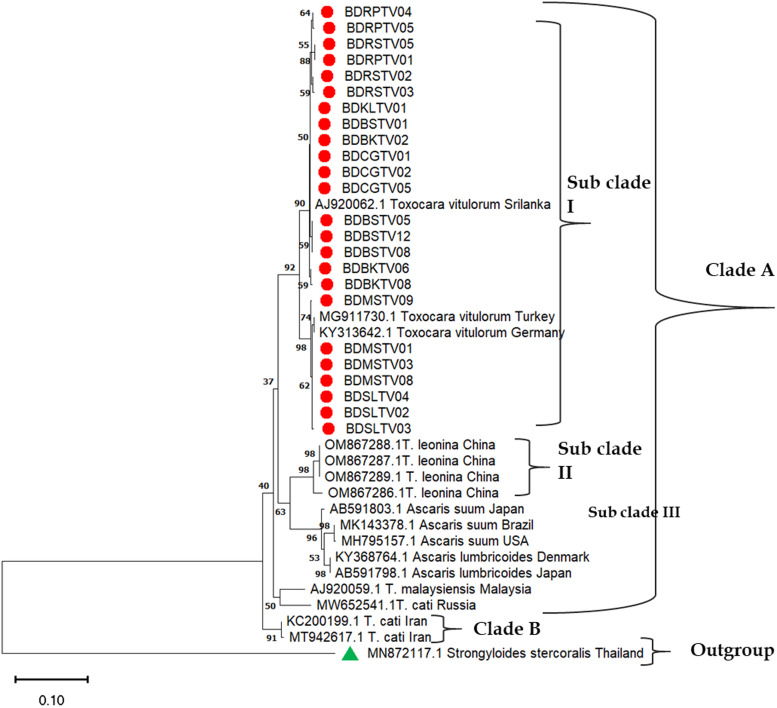
Neighbour-joining phylogenetic tree was constructed using partial *COX1* gene of *T. vitulorum* isolates from different hosts and geographical regions. *Strongyloides stercoralis* was used as an out group. Scale bar indicates the proportion of sites changing along each branch. [BD, Bangladesh; RP, Rangpur; RS, Rajshahi; CG, Chattogram; KL, Khulna; BS, Barishal; SL, Sylhet; MS, Mymensingh; TV, *Toxocara vitulorum*, the sur number was representative of isolate number].

**Figure 6.**
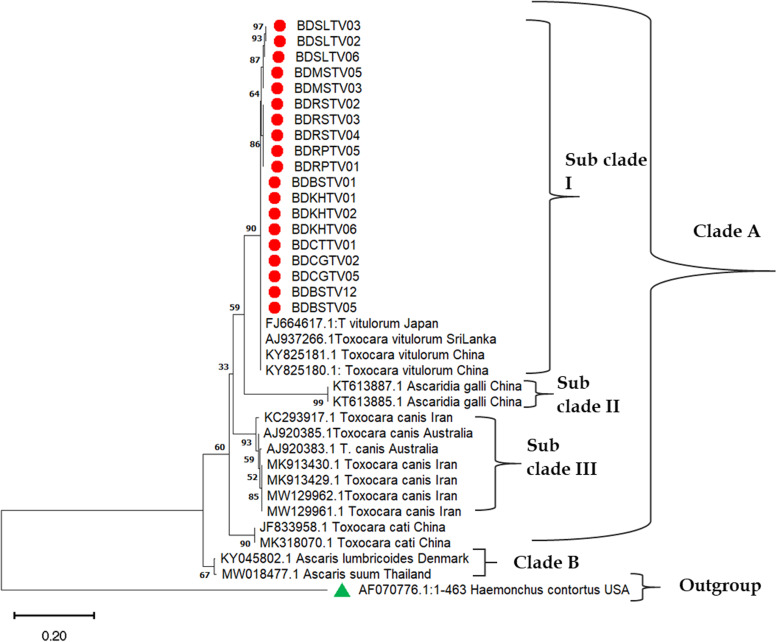
Neighbour-joining phylogenetic tree was constructed using partial *NAD1* gene of *T. vitulorum* isolates from different hosts and geographical regions. *H. contortus* was used as an out group. Scale bar indicates the proportion of sites changing along each branch. [BD, Bangladesh; RP, Rangpur; RS, Rajshahi; CG, Chattogram; KL, Khulna; BS, Barishal; SL, Sylhet; MS, Mymensingh; TV, *Toxocara vitulorum*, the sur number was representative of isolate number].

The phylogenetic trees were constructed using 24 *COX*1 and 21 *NAD1* gene sequences of *T. vitulorum* collected from 7 divisions of Bangladesh (Figs 5 and 6). Neighbour-Joining (NJ), Maximum Parsimony (MP) and Maximum Likelihood (ML) were used to produce phylogenetic tree that illustrate same findings. Readers better understanding, only the neighbour-joining trees have been incorporated, visualized and explained here. The NJ phylogeny for *COX1* gene generated with 1000 replicates showed two distinct clades A and B where clade A was further divided into three subclades. In subclade I, *T. vitulorum* isolates produced in this study were grouped together without any distinct boundary with Sri Lankan (AJ920062.1), German (KY313642.1), Turkish (MG911730.1) isolates and supported by strong bootstrap value (92%) (Fig. 5).

In case of NJ phylogeny for *NAD1* gene, two main clades A and B were also generated. In subclades I of clade A, *T. vitulorum* isolates were clustered with Japanese (FJ664617.1), Sri Lankan (AJ937266.1) and Chinese (KY825180.1 & KY825181.1) *T. vitulorum isolates*. The bootstrap value was 90% (Fig. 6).

### Population genetic structure

To evaluate genetic divergence among the examined populations, Fst and Nst values of *T. vitulorum* populations were calculated in various topographic zones of Bangladesh. When evaluating the *COX1* gene, genetic differentiation was observed and the pairwise Nst values ranged from −0.050 to 0.602 and Fst from −0.050 to 0.600. When compared populations from different topographic zones in Bangladesh, the *T. vitulorum* population of Barishal, Khulna and Rangpur exhibited rather substantial genetic divergence, with the greatest levels of Nst (0.602, 0.575, 0.559 to 0.9211) and Fst (0.600, 0.571 and 0.556) (Table 5).

**Table 5.** Gene flow and genetic differentiation indices between *T. vitulorum* genotypes based on *ITS2* region

Population 1	Population 2	Distance (km)	Hs	Ks	Kxy	Gst	DeltaSt	GammaSt	Nst	Fst	Dxy	Da
Mymensingh	Chattogram	400	0.889	3.714	7.667	0.089	0.007	0.479	0.567	0.565	0.021	0.012
Mymensingh	Barishal	292	0.889	3.714	8.333	0.089	0.008	0.510	0.602	0.600	0.023	0.014
Mymensingh	Khulna	330	1.000	4.286	9.000	0.003	0.008	0.487	0.559	0.556	0.025	0.014
Mymensingh	Rangpur	293	1.000	4.286	9.333	0.003	0.009	0.500	0.575	0.571	0.026	0.015
Mymensingh	Rajshahi	452	1.000	4.286	8.833	0.003	0.008	0.480	0.551	0.547	0.025	0.014
Mymensingh	Sylhet	279	1.000	4.571	9.167	0.003	0.008	0.470	0.531	0.527	0.026	0.014
Chattogram	Barishal	241	0.667	0.667	1.000	0.077	0.001	0.385	0.334	0.333	0.003	0.001
Chattogram	Khulna	442	0.833	1.333	2.000	0.032	0.002	0.385	0.334	0.333	0.006	0.002
Chattogram	Rangpur	545	0.833	1.333	2.000	0.032	0.002	0.385	0.334	0.333	0.006	0.002
Chattogram	Rajshahi	498	0.833	1.333	2.000	0.032	0.002	0.385	0.334	0.333	0.006	0.002
Chattogram	Sylhet	358	0.833	1.667	2.000	0.032	0.001	0.286	0.167	0.167	0.006	0.001
Barishal	Khulna	118	0.833	1.333	1.667	−0.034	0.001	0.304	0.201	0.200	0.005	0.001
Barishal	Rangpur	475	0.833	1.333	1.667	−0.034	0.001	0.304	0.201	0.200	0.005	0.001
Barishal	Rajshahi	323	0.833	1.333	1.667	−0.034	0.001	0.304	0.201	0.200	0.005	0.001
Barishal	Sylhet	400	0.833	1.667	1.444	−0.034	0.000	0.130	−0.155	−0.154	0.004	−0.001
Khulna	Rangpur	396	1.000	2.000	2.667	−0.059	0.002	0.333	0.252	0.250	0.007	0.002
Khulna	Rajshahi	258	1.000	2.000	2.667	−0.059	0.002	0.333	0.252	0.250	0.007	0.002
Khulna	Sylhet	424	1.000	2.333	2.222	−0.059	0.001	0.176	−0.050	−0.050	0.006	0.000
Rangpur	Rajshahi	227	1.000	2.000	2.667	−0.059	0.002	0.333	0.252	0.250	0.007	0.002
Rangpur	Sylhet	499	1.000	2.333	2.667	−0.059	0.002	0.263	0.126	0.125	0.007	0.001
Rajshahi	Sylhet	536	1.000	2.333	2.667	−0.059	0.002	0.263	0.126	0.125	0.007	0.001

Hs, Hudson's haplotype-based statistics; Ks, Hudson's nucleotide sequence-based statistics (Hudson *et al*., 1992); Kxy, Average proportion of nucleotide differences between *T. vitulorum* genotypes; Gst, Genetic differentiation index based on the frequency of haplotypes; Nst, Nucleotide-based statistics (Lynch and Crease, 1990); Fst, Tajima and Nei, pairwise genetic distance; Dxy, the average number of nucleotide substitutions per site between *T. vitulorum* genotypes; Da, the number of net nucleotide substitutions per site between *T. vitulorum* genotypes.

## Discussions

Parasite genomics is essential for determining epidemiology and controlling parasitic infections in humans and animals. *Toxocara vitulorum* is one of the most prevalent gastrointestinal helminths infecting ruminants, especially in tropical areas. *Toxocara vitulorum* has been found in several places in Bangladesh; however, there has never been an investigation into its molecular makeup and evolutionary status (Islam *et al*., 2005; Rahman *et al*., 2018). In this study, the population genetic structure and phylogenetic of *T. vitulorum* was investigated for the first time in Bangladesh. The findings obtained from the isolates of 7 divisions in Bangladesh and other global locations were evaluated, and their correlation was ascertained.

The present findings by morphological and morphometric measurements of body length and width, presence of 3 lips, spicules in male and finger like projections and straight posterior end in female *T. vitulorum* parasite are strongly supported by previous reports (Mahdy *et al*., 2020).

The variation of sequence identities among the *T. vitulorum* isolates was 3.0% in *ITS2* gene sequences. The degree of variation (3.0%) is comparable to that of isolates of with the variation of *T. vitulorum* from India, Sri Lanka, Egypt and Germany (Sultan *et al*., 2015; Venjakob *et al*., 2017). Twenty-nine distinct *ITS2* genotypes were detected among *T. vitulorum* isolates in present study, but the number of *ITS2* genotypes of *T. vitulorum* isolates were higher than described in previously reported (Sultan *et al*., 2015; Mahdy *et al*., 2020). The number of polymorphic loci of *T. vitulorum* isolates also differed between countries for as (Rast *et al*., 2013). Data obtained from this study confirmed the species as *T. vitulorum.*

The *ITS2, NAD1* and *COX1* genes were amplified from the genomic DNA of *T. vitulorum* species found in 7 divisions of Bangladesh in order to confirm the species' existence and investigate its molecular composition. The nucleotide BLAST search was used to retrieve best hit scoring *ITS2*, *COX1* and *NAD1* of *T. vitulorum* sequences with high identities (97–99%) from the GenBank and ClustalW program in Mega 11 was used to align all the sequences (Ahmed *et al*., 2023). For the *COX1* and *NAD1* gene sequences, a total of 12 and 6 distinct haplotypes were found respectively. Five SNPs were discovered in the *NAD1* gene, while 18 SNPs were found in the *COX1* gene. A great amount of gene flow of *COX1* and *NAD1* gene were observed among *T. vitulorum* species of Bangladesh and for *COX1*, the average nucleotide diversity was 0.01691 and haplotype diversity was 0.89493 for *COX1* and for *NAD1* average nucleotide diversity was 0.00658 and haplotype diversity was 0.77895 respectively. In comparison to previously published estimates of nucleotide diversity from *T. vitulorum* isolates in Sri Lanka, the recorded values for both cases are more scattered. (Wickramasinghe *et al*., 2009*a*, 2009*b*), India (Mahdy *et al*., 2020), Egypt (Sultan *et al*., 2015) and Turkey (Oguz, 2018).

The topology of the *ITS2* phylogenetic tree showed two distinct clades, clade A and B. Clade A was again divided into subclade I and subclade II when compared with the isolates of this study with those of other countries. Subclade I representing all the 29 *T. vitulorum* genotypes showed little resolution and belonged to samples isolated from 7 districts of Bangladesh along with other *T. vitulorum* genotypes including India (MK100346.1 &KJ777159.1), Egypt (MG214151.1), Germany (KY442062.1), Canada (JQ083352.1), Sri Lanka (FJ418784.1), USA (KT3738.1) and *T. cati* from India (KJ777179), China (KY003088), Italy (MZ59634.1) and Japan (AB571303) that had been consistent with previously published reports (Wickramasinghe *et al*., 2009*a*, 2009*b*).

The NJ dendrogram for *COX1* gene generated with 1000 replicates illustrates 2 definite clades A and B where clade A was further divided into 3 subclades. In subclade I, *T. vitulorum* isolates produced in this study grouped together without any distinct boundary with Sri Lankan (AJ920062.1), German (KY313642.1), Turkey (MG911730.1) isolates, were supported by the strong bootstrap value (92%) and documented the previously published reports (Oguz, 2018; Mahdy *et al*., 2020). In case of NJ phylogeny for *NAD1* gene, 2 main clades A and B were also generated. In subclades I of clade A, *T. vitulorum* isolates were clustered with Japanese (FJ664617.1), Sri Lankan (AJ937266.1) and Chinese (KY825180.1 & KY825181.1) *T. vitulorum* isolates. The results coincide with the same attributes that previously published (Li *et al*., 2016).

In order to ascertain genetic variation within the examined *Toxocara* populations, *T. vitulorum* populations' Fst and Nst values were calculated across various Bangladeshi topography zones. The pairwise FST values were recorded more than 0.5 when the population of Mymensingh compared with populations from all other 6 divisions. The highest Fst were seen between *T. vitulorum* populations of Mymensingh and Barishal zone followed by Mymensingh-Rangpur, Mymensingh-Khulna and Mymensingh-Chattogram zone. It was further bolstered by the presence of a very low level of gene flow between them. The low level of gene flow may in part be due to lack of prenatal and transmammary transmission in case of *T. vitulorum* infection. The horizontal gene flow occurs due to the movement of infected felids, whereas the vertical gene flow occurs due to transmammary transmission and limited gene flow between populations can expedite the process of genetic differentiation (Choy *et al*., 2015). The results are consistent with previously published data for Nst (Oguz, 2018) whereas much higher than *T. canis* population isolated from different regions of Iran (Ozlati *et al*., 2016) and lower than *T. cati* populations in China (Venkatesan *et al*., 2022). All the above results suggest that high genetic differentiation and low gene flow without clear geographical barriers and cross-infection between population of this certain area is not frequently occurred. We hypothesize that random movement of the buffalo calves act as the medium for genetic exchange. Thus, further investigations are warranted to take into account in the design of an effective control strategy.

## Supporting information

Biswas et al. supplementary material 1 Biswas et al. supplementary material

Biswas et al. supplementary material 2 Biswas et al. supplementary material

Biswas et al. supplementary material 3 Biswas et al. supplementary material

Biswas et al. supplementary material 4 Biswas et al. supplementary material

## Data Availability

Data will be available based on request.

## Supplementary material

The supplementary material for this article can be found at https://doi.org/10.1017/S0031182024000842.
