# Supplementary material for: Molecular characterization and genetic variability of Toxocara vitulorum from naturally infected buffalo calves for the first time in Bangladesh
Source: Parasitology. 2024 Oct 15;151(8):795–807. doi: 10.1017/S0031182024000842 (PMC11579036; doi:10.1017/S0031182024000842)
Supplement: Biswas et al. supplementary material 1 — Biswas et al. supplementary material [file S0031182024000842sup001.docx]

Table S1: Pairwise nucleotidic genetic distances (p-distance model) of the partial *ITS2* sequences for *T. vitulorum* isolates in the present study with best hit scoring reference sequences of different countries retrieved from GenBank

| ***ITS2* Sequences** | **1** | **2** | **3** | **4** | **5** | **6** | **7** | **8** | **9** | **10** | **11** | **12** | **13** | **14** | **15** | **16** | **17** | **18** | **19** | **20** | **21** | **22** | **23** | **24** | **25** | **26** | **27** | **28** | **29** | **30** | **31** | **32** | **33** | **34** |
| --- | --- | --- | --- | --- | --- | --- | --- | --- | --- | --- | --- | --- | --- | --- | --- | --- | --- | --- | --- | --- | --- | --- | --- | --- | --- | --- | --- | --- | --- | --- | --- | --- | --- | --- |
| BDBSTV01 | - |  |  |  |  |  |  |  |  |  |  |  |  |  |  |  |  |  |  |  |  |  |  |  |  |  |  |  |  |  |  |  |  |  |
| BDBSTV05 | 0.00 | - |  |  |  |  |  |  |  |  |  |  |  |  |  |  |  |  |  |  |  |  |  |  |  |  |  |  |  |  |  |  |  |  |
| BDBSTV08 | 0.01 | 0.01 | - |  |  |  |  |  |  |  |  |  |  |  |  |  |  |  |  |  |  |  |  |  |  |  |  |  |  |  |  |  |  |  |
| BDBSTV12 | 0.02 | 0.02 | 0.01 | - |  |  |  |  |  |  |  |  |  |  |  |  |  |  |  |  |  |  |  |  |  |  |  |  |  |  |  |  |  |  |
| BDCGTV01 | 0.00 | 0.00 | 0.01 | 0.03 | - |  |  |  |  |  |  |  |  |  |  |  |  |  |  |  |  |  |  |  |  |  |  |  |  |  |  |  |  |  |
| BDCGTV02 | 0.00 | 0.00 | 0.01 | 0.03 | 0.00 | - |  |  |  |  |  |  |  |  |  |  |  |  |  |  |  |  |  |  |  |  |  |  |  |  |  |  |  |  |
| BDCGTV05 | 0.00 | 0.00 | 0.01 | 0.03 | 0.00 | 0.00 | - |  |  |  |  |  |  |  |  |  |  |  |  |  |  |  |  |  |  |  |  |  |  |  |  |  |  |  |
| BDCGTV07 | 0.00 | 0.00 | 0.01 | 0.03 | 0.00 | 0.00 | 0.00 | - |  |  |  |  |  |  |  |  |  |  |  |  |  |  |  |  |  |  |  |  |  |  |  |  |  |  |
| BDKLTV08 | 0.00 | 0.00 | 0.01 | 0.02 | 0.00 | 0.00 | 0.00 | 0.00 | - |  |  |  |  |  |  |  |  |  |  |  |  |  |  |  |  |  |  |  |  |  |  |  |  |  |
| BDKLTV01 | 0.00 | 0.00 | 0.01 | 0.02 | 0.00 | 0.00 | 0.00 | 0.00 | 0.00 | - |  |  |  |  |  |  |  |  |  |  |  |  |  |  |  |  |  |  |  |  |  |  |  |  |
| BDKLTV02 | 0.00 | 0.00 | 0.01 | 0.03 | 0.01 | 0.01 | 0.01 | 0.01 | 0.00 | 0.00 | - |  |  |  |  |  |  |  |  |  |  |  |  |  |  |  |  |  |  |  |  |  |  |  |
| BDKLTV06 | 0.00 | 0.00 | 0.01 | 0.02 | 0.00 | 0.00 | 0.00 | 0.00 | 0.00 | 0.00 | 0.00 | - |  |  |  |  |  |  |  |  |  |  |  |  |  |  |  |  |  |  |  |  |  |  |
| BDMSTV01 | 0.01 | 0.01 | 0.02 | 0.04 | 0.02 | 0.02 | 0.02 | 0.02 | 0.01 | 0.01 | 0.02 | 0.01 | - |  |  |  |  |  |  |  |  |  |  |  |  |  |  |  |  |  |  |  |  |  |
| BDMSTV03 | 0.01 | 0.01 | 0.02 | 0.04 | 0.02 | 0.02 | 0.02 | 0.02 | 0.01 | 0.01 | 0.02 | 0.01 | 0.00 | - |  |  |  |  |  |  |  |  |  |  |  |  |  |  |  |  |  |  |  |  |
| BDMSTV04 | 0.01 | 0.01 | 0.02 | 0.04 | 0.02 | 0.02 | 0.02 | 0.02 | 0.01 | 0.01 | 0.02 | 0.01 | 0.00 | 0.00 | - |  |  |  |  |  |  |  |  |  |  |  |  |  |  |  |  |  |  |  |
| BDMSTV08 | 0.01 | 0.01 | 0.02 | 0.04 | 0.02 | 0.02 | 0.02 | 0.02 | 0.01 | 0.01 | 0.02 | 0.01 | 0.00 | 0.00 | 0.00 | - |  |  |  |  |  |  |  |  |  |  |  |  |  |  |  |  |  |  |
| BDMSTV09 | 0.02 | 0.02 | 0.03 | 0.05 | 0.03 | 0.03 | 0.03 | 0.03 | 0.02 | 0.02 | 0.03 | 0.02 | 0.01 | 0.01 | 0.01 | 0.01 | - |  |  |  |  |  |  |  |  |  |  |  |  |  |  |  |  |  |
| BDSLTV02 | 0.02 | 0.02 | 0.03 | 0.04 | 0.03 | 0.03 | 0.03 | 0.03 | 0.02 | 0.02 | 0.03 | 0.02 | 0.01 | 0.01 | 0.01 | 0.01 | 0.02 | - |  |  |  |  |  |  |  |  |  |  |  |  |  |  |  |  |
| BDSLTV03 | 0.02 | 0.02 | 0.03 | 0.04 | 0.03 | 0.03 | 0.03 | 0.03 | 0.02 | 0.02 | 0.03 | 0.02 | 0.01 | 0.01 | 0.01 | 0.01 | 0.02 | 0.00 | - |  |  |  |  |  |  |  |  |  |  |  |  |  |  |  |
| BDSLTV04 | 0.03 | 0.03 | 0.04 | 0.05 | 0.03 | 0.03 | 0.03 | 0.03 | 0.03 | 0.03 | 0.03 | 0.03 | 0.01 | 0.01 | 0.01 | 0.01 | 0.02 | 0.00 | 0.00 | - |  |  |  |  |  |  |  |  |  |  |  |  |  |  |
| BDSLTV05 | 0.02 | 0.02 | 0.03 | 0.04 | 0.03 | 0.03 | 0.03 | 0.03 | 0.02 | 0.02 | 0.03 | 0.02 | 0.01 | 0.01 | 0.01 | 0.01 | 0.02 | 0.00 | 0.00 | 0.00 | - |  |  |  |  |  |  |  |  |  |  |  |  |  |
| BDRSTV02 | 0.00 | 0.00 | 0.01 | 0.02 | 0.00 | 0.00 | 0.00 | 0.00 | 0.00 | 0.00 | 0.00 | 0.00 | 0.01 | 0.01 | 0.01 | 0.01 | 0.02 | 0.02 | 0.02 | 0.03 | 0.02 | - |  |  |  |  |  |  |  |  |  |  |  |  |
| BDRSTV03 | 0.00 | 0.00 | 0.01 | 0.02 | 0.00 | 0.00 | 0.00 | 0.00 | 0.00 | 0.00 | 0.00 | 0.00 | 0.01 | 0.01 | 0.01 | 0.01 | 0.02 | 0.02 | 0.02 | 0.03 | 0.02 | 0.00 | - |  |  |  |  |  |  |  |  |  |  |  |
| BDRSTV05 | 0.00 | 0.00 | 0.01 | 0.02 | 0.00 | 0.00 | 0.00 | 0.00 | 0.00 | 0.00 | 0.00 | 0.00 | 0.01 | 0.01 | 0.01 | 0.01 | 0.02 | 0.02 | 0.02 | 0.03 | 0.02 | 0.00 | 0.00 | - |  |  |  |  |  |  |  |  |  |  |
| BDRSTV06 | 0.00 | 0.00 | 0.01 | 0.02 | 0.00 | 0.00 | 0.00 | 0.00 | 0.00 | 0.00 | 0.00 | 0.00 | 0.01 | 0.01 | 0.01 | 0.01 | 0.02 | 0.02 | 0.02 | 0.03 | 0.02 | 0.00 | 0.00 | 0.00 | - |  |  |  |  |  |  |  |  |  |
| BDRPTV01 | 0.00 | 0.00 | 0.01 | 0.02 | 0.01 | 0.01 | 0.01 | 0.01 | 0.00 | 0.00 | 0.01 | 0.00 | 0.02 | 0.02 | 0.02 | 0.02 | 0.03 | 0.02 | 0.02 | 0.03 | 0.02 | 0.00 | 0.00 | 0.00 | 0.00 | - |  |  |  |  |  |  |  |  |
| BDRPTV04 | 0.00 | 0.00 | 0.01 | 0.02 | 0.00 | 0.00 | 0.00 | 0.00 | 0.00 | 0.00 | 0.00 | 0.00 | 0.01 | 0.01 | 0.01 | 0.01 | 0.02 | 0.02 | 0.02 | 0.03 | 0.02 | 0.00 | 0.00 | 0.00 | 0.00 | 0.00 | - |  |  |  |  |  |  |  |
| BDRPTV05 | 0.00 | 0.00 | 0.01 | 0.02 | 0.00 | 0.00 | 0.00 | 0.00 | 0.00 | 0.00 | 0.00 | 0.00 | 0.01 | 0.01 | 0.01 | 0.01 | 0.02 | 0.02 | 0.02 | 0.03 | 0.02 | 0.00 | 0.00 | 0.00 | 0.00 | 0.00 | 0.00 | - |  |  |  |  |  |  |
| BDRPTV06 | 0.00 | 0.00 | 0.01 | 0.02 | 0.00 | 0.00 | 0.00 | 0.00 | 0.00 | 0.00 | 0.00 | 0.00 | 0.01 | 0.01 | 0.01 | 0.01 | 0.02 | 0.02 | 0.02 | 0.03 | 0.02 | 0.00 | 0.00 | 0.00 | 0.00 | 0.00 | 0.00 | 0.00 | - |  |  |  |  |  |
| MK100346.1*T. vitulorum* Nagaland | 0.00 | 0.00 | 0.01 | 0.02 | 0.00 | 0.00 | 0.00 | 0.00 | 0.00 | 0.00 | 0.00 | 0.00 | 0.01 | 0.01 | 0.01 | 0.01 | 0.02 | 0.02 | 0.02 | 0.03 | 0.02 | 0.00 | 0.00 | 0.00 | 0.00 | 0.00 | 0.00 | 0.00 | 0.00 | - |  |  |  |  |
| MG214152.1 *T. vitulorum* Egypt | 0.00 | 0.00 | 0.01 | 0.02 | 0.00 | 0.00 | 0.00 | 0.00 | 0.00 | 0.00 | 0.00 | 0.00 | 0.01 | 0.01 | 0.01 | 0.01 | 0.02 | 0.02 | 0.02 | 0.03 | 0.02 | 0.00 | 0.00 | 0.00 | 0.00 | 0.00 | 0.00 | 0.00 | 0.00 | 0.00 | - |  |  |  |
| FJ418784.1 *T. vitulorum* Sri Lanka | 0.00 | 0.00 | 0.01 | 0.03 | 0.01 | 0.01 | 0.01 | 0.01 | 0.00 | 0.00 | 0.01 | 0.00 | 0.02 | 0.02 | 0.02 | 0.02 | 0.03 | 0.03 | 0.03 | 0.03 | 0.03 | 0.00 | 0.00 | 0.00 | 0.00 | 0.01 | 0.00 | 0.00 | 0.00 | 0.00 | 0.00 | - |  |  |
| MG214151.1 *T. vitulorum* Egypt | 0.00 | 0.00 | 0.01 | 0.03 | 0.01 | 0.01 | 0.01 | 0.01 | 0.00 | 0.00 | 0.01 | 0.00 | 0.02 | 0.02 | 0.02 | 0.02 | 0.03 | 0.03 | 0.03 | 0.03 | 0.03 | 0.00 | 0.00 | 0.00 | 0.00 | 0.01 | 0.00 | 0.00 | 0.00 | 0.00 | 0.00 | 0.00 | - |  |
| KY442062.1*T. vitulorum* Germany | 0.00 | 0.00 | 0.01 | 0.03 | 0.01 | 0.01 | 0.01 | 0.01 | 0.00 | 0.00 | 0.01 | 0.00 | 0.02 | 0.02 | 0.02 | 0.02 | 0.03 | 0.03 | 0.03 | 0.03 | 0.03 | 0.00 | 0.00 | 0.00 | 0.00 | 0.01 | 0.00 | 0.00 | 0.00 | 0.00 | 0.00 | 0.00 | 0.00 | 0.00 |

[Note: BD=Bangladesh, RP= Rangpur, RS=Rajshahi, CG=Chattogram, KL= Khulna, BS=Barishal, SL=Sylhet, MS=Mymensingh, TV=*Toxocara vitulorum*, the sur number was representative of isolate number]
