# Supplementary material for: Molecular characterization and genetic variability of Toxocara vitulorum from naturally infected buffalo calves for the first time in Bangladesh
Source: Parasitology. 2024 Oct 15;151(8):795–807. doi: 10.1017/S0031182024000842 (PMC11579036; doi:10.1017/S0031182024000842)
Supplement: Biswas et al. supplementary material 4 — Biswas et al. supplementary material [file S0031182024000842sup004.docx]

Table S4: List of GenBank-retrieved sequences that were used to construct phylogenetic trees in this study

| **Gene** | **Species** | **Host** | **Country** | **Accession no. in GenBank** |
| --- | --- | --- | --- | --- |
| *ITS2* | *T. vitulorum* | Buffalo | India | MK100346.1 |
|  | *T. vitulorum* | Buffalo | Egypt | MG214152.1 |
|  | *T. vitulorum* | Buffalo | India | KJ777159.1 |
|  | *T. vitulorum* | Buffalo | USA | FJ418784.1 |
|  | *T. vitulorum* | Buffalo | Canada | JQ083352.1 |
|  | *T. vitulorum* | Buffalo | Egypt | MG214151.1 |
|  | *T. vitulorum* | Buffalo | Germany | KY442062.1 |
|  | *T. cati* | Cat | Sri Lanka | AJ002440.1 |
|  | *T. cati* | Cat | India | OK668294.1 |
|  | *T. cati* | Cat | China | KY003088.1 |
|  | *T. cati* | Cat | India | KJ777179.1 |
|  | *T. cati* | Cat | Japan | AB571303.1 |
|  | *T. cati* | Cat | Japan | AB110033.1 |
|  | *T. cati* | Cat | Italy | MZ596341.1 |
|  | *T. canis* | Dog | Egypt | MG214150.1 |
|  | *T. canis* | Dog | India | OK635791.1 |
|  | *T. canis* | Dog | Sri Lanka | FJ418788.1 |
|  | *T. canis* | Dog | Iran | AB743615.1 |
| *COX1* | *T. vitulorum* | Buffalo | Sri Lanka | AJ920062.1 |
|  | *T. vitulorum* | Buffalo | Turkey | MG911730.1 |
|  | *T. vitulorum* | Buffalo | Germany | KY313642.1 |
|  | *T. leonina* | DOG/CAT | China | OM867288.1 |
|  | *T. leonina* | DOG/CAT | China | OM867287.1 |
|  | *T. leonina* | DOG/CAT | China | OM867289.1 |
|  | *T. leonina* | DOG/CAT | China | 0M867286.1 |
|  | *Ascaris suum* | Swine | Japan | AB591803.1 |
|  | *Ascaris suum* | Swine | Brazil | MK143378.1 |
|  | *Ascaris suum* | Swine | USA | MH795157.1 |
|  | *Ascaris lumbricoides* | Human | Denmark | KY368764.1 |
|  | *Ascaris lumbricoides* | Human | Japan | AB591798.1 |
|  | *T. malaysiensis* | Cat | Malaysia | AJ920059.1 |
|  | *T. cati* | Cat | Russia | MW652541.1 |
|  | *T. cati* | Cat | Iran | KC200199.1 |
|  | *T. cati* | Cat | Iran | MT942617.1 |
| *NAD1* | *T. vitulorum* | Buffalo | Japan | FJ664617.1 |
|  | *T. vitulorum* | Buffalo | Sri Lanka | AJ937266.1 |
|  | *T. vitulorum* | Buffalo | China | KY825180.1 |
|  | *T. vitulorum* | Buffalo | China | KY825181.1 |
|  | *Ascaridia gali* | Chicken | China | KT613885.1 |
|  | *T. canis* | Dog | China | KT613887.1 |
|  | *T. canis* | Dog | Iran | KC293917.1 |
|  | *T. canis* | Dog | Australia | AJ920383.1 |
|  | *T. canis* | Dog | Australia | AJ920385.1 |
|  | *T. canis* | Dog | Iran | MK913429.1 |
|  | *T. canis* | Dog | Iran | MK913430.1 |
|  | *T. canis* | Dog | Iran | MW129961.1 |
|  | *T. canis* | Dog | Iran | MW129962.1 |
|  | *T. cati* | Cat | China | JF833958.1 |
|  | *T. cati* | Cat | China | MK318070.1 |
|  | *Ascaris lumbricoides* | Human | Denmark | KY045802.1 |
|  | *Ascaris suum* | Pig | Thailand | MW018477.1 |
